# Nationwide Big Data Analysis of Statin Use and Intracerebral Hemorrhage Risk in Acute Ischemic Stroke Patients in Taiwan

**DOI:** 10.3390/medicina60060939

**Published:** 2024-06-04

**Authors:** William Winardi, Sin-Hua Moi, Thomas Winardi, Yu-Wen Cheng, Po-Yuan Chen, Cheng-Kai Lin

**Affiliations:** 1 School of Medicine, College of Medicine, I-Shou University, Kaohsiung 84001, Taiwan; winardi930@yahoo.com; 2 Department of Neurosurgery, E-Da Hospital, Kaohsiung 82445, Taiwan; 3 Graduate Institute of Clinical Medicine, College of Medicine, Kaohsiung Medical University, Kaohsiung 80708, Taiwan; moish@kmu.edu.tw; 4 Department of Medical Research, Kaohsiung Medical University, Kaohsiung 80708, Taiwan; 5 Research Center for Precision Environmental Medicine, Kaohsiung Medical University, Kaohsiung 80708, Taiwan; 6 Nicolaus Copernicus University, 87100 Toruń, Poland; 7 Department of Neurosurgery, Kaohsiung Veterans General Hospital, Kaohsiung 81362, Taiwan; murraycheng1015@gmail.com

**Keywords:** statins, ischemic stroke, intracerebral hemorrhage, mortality, dosage

## Abstract

*Background and Objectives*: Although statins are recommended for secondary prevention of acute ischemic stroke, some population-based studies and clinical evidence suggest that they might be used with an increased risk of intracranial hemorrhage. In this nested case–control study, we used Taiwan’s nationwide universal health insurance database to investigate the possible association between statin therapy prescribed to acute ischemic stroke patients and their risk of subsequent intracerebral hemorrhage and all-cause mortality in Taiwan. *Materials and Methods*: All data were retrospectively obtained from Taiwan’s National Health Insurance Research Database. Acute ischemic stroke patients were divided into a cohort receiving statin pharmacotherapy and a control cohort not receiving statin pharmacotherapy. A 1:1 matching for age, gender, and index day, and propensity score matching was conducted, producing 39,366 cases and 39,366 controls. The primary outcomes were long-term subsequent intracerebral hemorrhage and all-cause mortality. The competing risk between subsequent intracerebral hemorrhage and all-cause mortality was estimated using the Fine and Gray regression hazards model. *Results*: Patients receiving statin pharmacotherapy after an acute ischemic stroke had a significantly lower risk of subsequent intracerebral hemorrhage (*p* < 0.0001) and lower all-cause mortality rates (*p* < 0.0001). Low, moderate, and high dosages of statin were associated with significantly decreased risks for subsequent intracerebral hemorrhage (adjusted sHRs 0.82, 0.74, 0.53) and all-cause mortality (adjusted sHRs 0.75, 0.74, 0.74), respectively. *Conclusions*: Statin pharmacotherapy was found to safely and effectively reduce the risk of subsequent intracerebral hemorrhage and all-cause mortality in acute ischemic stroke patients in Taiwan.

## 1. Introduction

Stroke ranks as the second-leading cause of mortality and the third-leading cause of mortality and disability in the world [1]. In 2019, the number of acute ischemic stroke (AIS) cases and stroke-related deaths were 7,630,803 cases with a crude incidence of 94.51 and 42.56 per 100,000 per year (95% uncertainty interval (UI): 81.91–110.76 95 and 95%UI: 38.43–45.70, respectively) [1]. In Taiwan, where more than 12,000 people die from an AIS yearly, stroke ranks as the fourth leading cause of death [2]. Based on these data and respective population sizes, the incidence of AIS in Taiwan is more than half that of the global incidence.

The management of AIS in Taiwan generally adheres to the 2019 updated Guidelines for the Early Management of Patients with Acute Ischemic Stroke [3]. Those guidelines focus on the control of hyperlipidemia, which includes lifestyle changes such as control of sodium, glucose, and lipid intake, as well as pharmacological control, particularly high-intensity statin treatment modified for at-risk groups [3]. In Taiwan, these guidelines may be modified by physicians based on their personal experience and the National Health Insurance criteria for reimbursement. 

Although statins are currently recommended by the American Heart Association (AHA)/American Stroke Association for secondary prevention of AIS [3], various meta-analyses have come to different conclusions regarding the use of statin pharmacotherapy after AIS [4,5,6]. The Stroke Prevention by Aggressive Reduction in Cholesterol Levels trial (SPARCL) found that patients receiving high-dose atorvastatin therapy had a higher incidence of subsequent intracranial hemorrhage (sICH) than those not receiving this therapy (2.3% vs. 1.4%) [7]. One sequential analysis of randomized controlled trials totaling 9938 patients found a significant increase in sICH in those taking statins (RR = 1.4, 95%CI 1.04–1.89, *p* = 0.03) [4]. However, that analysis only included one trial conducted in Asia, notably Japan, a study that found no significant difference [4]. One population study from Korea also reported a lower incidence of subsequent sICH [8]. These studies do not provide enough data to conclude that there are interethnic differences in the risk of sICH in patients taking statins. This study used Taiwan’s National Health Insurance Research Database (NHIRD) to investigate the relationship between nationwide statin prescriptions and sICH and all-cause mortality. 

## 2. Materials and Methods

### 2.1. Data Source

All data were obtained from Taiwan’s NHIRD, a national health insurance database managed by Taiwan’s Ministry of Health and Welfare. The NHIRD contains all diagnoses and insurance claims for medical services and prescriptions for nearly all of Taiwan’s 23.57 million residents, making up more than 99% of the population. We collected claims for all medications prescribed for all outpatients and inpatients between 2000 and 2017. Patient identification numbers were encrypted before the release of the database to ensure patient confidentiality. Details regarding the database generation, monitoring, and maintenance are provided on the NHIRD website (http://nhird.nhri.org.tw/) (Accessed on 1 August 2019). This study was approved by the Institutional Review Board of E-Da Hospital (EMRP-10-061).

### 2.2. Study Cohort

**Figure 1:**
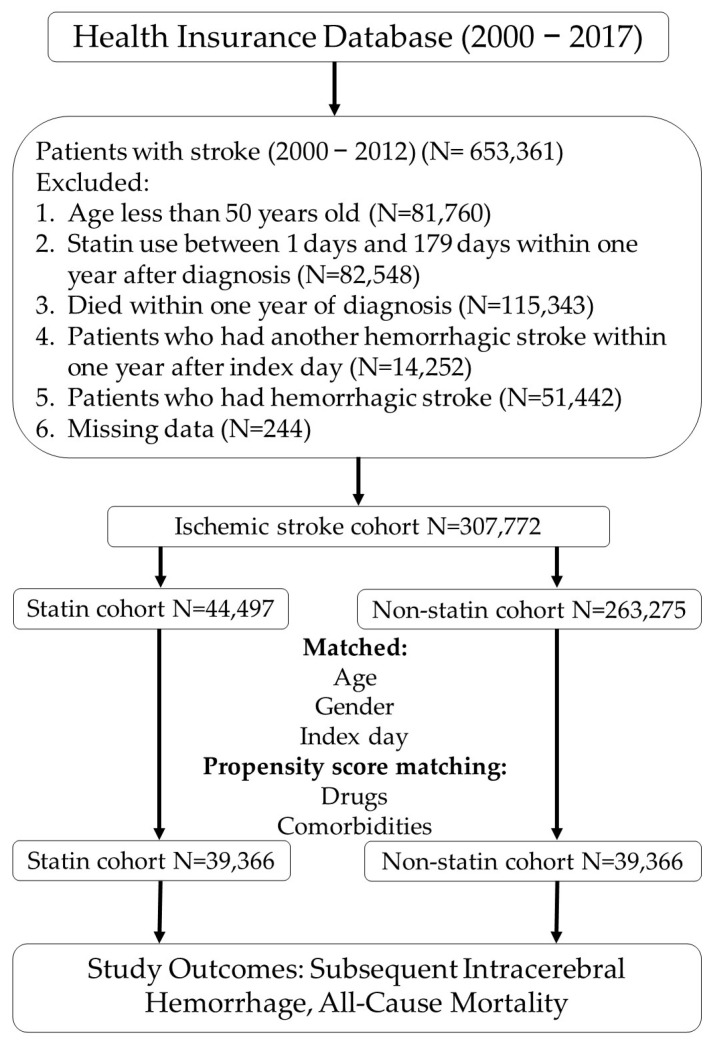
Flow chart of the study population.

At the time of data collection, the NHIRD classified diagnoses using the International Classification of Diseases, 9th Revision—Clinical Modification (ICD-9-CM). As can be seen in our flow chart for this retrospective nested case–control study (Figure 1), we enrolled patients diagnosed as having an ischemic stroke (433.X and 434.X) and intracerebral hemorrhage (431.X) based on their hospitalization discharge records from 1 January 2000 to 31 December 2012. In total, we identified 653,361 stroke patients. We excluded patients below 50 years old due to concern over young stroke, a disease that may have different etiologies from stroke in older patients [9]. We also excluded patients who had received statins between 1 and 179 days after the index date, which we defined as the date of initial AIS diagnosis; those who had died one year within the index date; and those who had another intracranial hemorrhage (ICH) within one year of index date to ensure the statins had had enough time to exert their clinical effects. Finally, we excluded patients who had had hemorrhagic strokes as well as those with incomplete data. After exclusion, we were left with 307,772 patients to include in our analysis. The AIS patients were assigned to statin and non-statin cohorts. Patient comorbidities before the index date were classified and analyzed using the Charlson Comorbidity Index (CCI). The end of follow-up for each patient was defined as the day of withdrawal from the NHI program, death, or the end of our study period. We collected at least five years of follow-up data for each AIS patient. After individual 1:1 matching for age, gender, and index day and propensity score matching for medications and comorbidities, we were left with 39,366 patients to be enrolled in the statin cohort and 39,366 to be enrolled in the non-statin cohort.

### 2.3. Study Outcomes

We used ICD-9-CM: 431.X to identify patients diagnosed with ICH in the NHIRD and determine the incidence of the disease and cause of death. The data collected from the NHIRD were then linked to the NHIRD death registration data, a separate database also managed by Taiwan’s Ministry of Health and Welfare. The causes of death we were particularly interested in were the 17 disease entities contributing to the Charlson Comorbidity Index and identified by ICD-9-CM codes. Statin dosage was defined as high, moderate, or low following the 2018 American College of Cardiology/AHA Classification of Intensity [10]. 

The primary outcome of this study was subsequent intracerebral hemorrhage (sICH), defined as a diagnosis of ICH at admission one year after the index date, a time period making it more likely that the sICH would be statin-related. The secondary outcome was all-cause mortality, defined as withdrawal from the NHI program, which would be indicated in the database. All patients were followed until death or the end of the study period (31 December 2017).

### 2.4. Study Variables

Potential confounding factors included age; gender; statin dose; duration of statin pharmacotherapy; concurrent medication use; including thrombolytics, anticoagulants, antihypertensives, non-steroidal anti-inflammatory drugs (NSAIDs), and selective serotonin reuptake inhibitors (SSRIs); and Charlson comorbidities identified by their respective ICD-9-CM codes (Table S1).

### 2.5. Statistical Analyses

Baseline characteristics and comorbidities for the statin and the non-statin cohorts are summarized descriptively. Chi-squared or Student’s *t*-tests were used to analyze differences between the two cohorts. In our effort to rigorously calculate the risk of sICH, we considered death as a competing event. The Fine and Gray regression model was used to calculate the sub-distribution hazard ratio (sHR). *p*-values were calculated by Gray’s test. 

The Cox proportional hazards model was applied to calculate cumulative survival hazard ratio (HR) analysis, adjusting for age; gender; pharmacotherapy with thrombolytic agents, anticoagulant agents, antihypertensive agents, NSAIDs, and SSRIs; and Charlson comorbidities. The Kaplan–Meier method was used to determine the cumulative incidence of sICH and the probability of survival. Cohort differences were analyzed by log-rank test. All data were managed, and all statistical operations were performed using the PHREG package and SAS version 9.4.

## 3. Results

### 3.1. Baseline Characteristics

The baseline characteristics of patients identified as having had AIS and taking statin are presented alongside the matched cohorts in Table 1. After exclusion, 307,772 patients 50 years old and older were diagnosed as having AIS between 1 January 2000 and 31 December 2012. These patients were assigned to statin and non-statin cohorts matched for age, gender, index day, and propensity score matching for medication usage and comorbidities, each cohort containing 39,366 patients. A total of 12,318 received low-intensity statin pharmacotherapy for 294.84 ± 77.72 days; 21,729 received moderate-intensity statin pharmacotherapy for 242.70 ± 112.38 days; and 5319 received high-intensity statin pharmacotherapy for 209.32 ± 129.71 days. 

Although we had performed 1:1 matching for age, gender, and index day, as well as propensity score matching for medications and comorbidities, we still found some differences between the non-statin and statin cohorts in our real-world data. Significantly more patients in the statin cohort were treated with SSRIs compared to those in the non-statin cohort (*p* = 0.0077); fewer than three patients received thrombolytics; and significantly more patients in the statin cohort had the following comorbidities: myocardial infarction (*p* < 0.0001), congestive heart failure (*p* = 0.0034), peripheral vascular disease (*p* = 0.0265), cerebrovascular disease (*p* < 0.0001), dementia (*p* < 0.0001), chronic lung disease (*p* < 0.0001), and moderate or severe kidney disease (*p* < 0.0105). However, in the non-statin cohort, we found that more patients had been prescribed antihypertensive pharmacotherapy compared to the statin cohort (*p* = 0.0401) and that they had significantly more chronic liver diseases, ICH diagnoses, and all-cause mortality rate (all *p* < 0.0001) (Table 1).

### 3.2. Statin Use and Subsequent ICH

**Figure 2:**
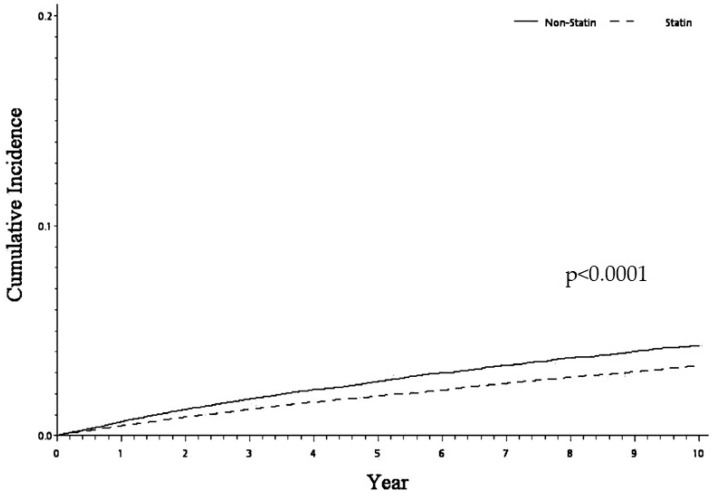
Cumulative incidence of hemorrhagic stroke between statin and non-statin cohorts for patients receiving statin treatment after ischemic stroke.

The sHR for sICH risk was calculated using the Fine and Gray’s model. As can be seen in Table 2, after adjustment for age and gender, statin users were at lower risk of sICH (sHR = 0.74, 95%CI: 0.68–0.80, *p* < 0.0001) and had a lower cumulative incidence of the disease (*p* < 0.0001) (Figure 2). It should also be noted that patients who had peptic ulcer disease also had a lower risk of sICH (sHR = 0.82, 95%CI: 0.74–0.90, *p* < 0.0001) (Table 2).

As can also be seen in Table 2, the only pharmacotherapeutic risk factor independently associated with sICH in this study was anticoagulant use (sHR = 1.45, 95%CI: 1.24–1.70, *p* < 0.0001). The other independent risk factors for sICH were comorbid cerebrovascular disease (sHR = 1.18, 95%CI: 1.08–1.28, *p* = 0.0002), diabetes with end-organ damage (sHR = 1.19, 95%CI: 1.05–1.35, *p* = 0.0067), hemiplegia (sHR = 1.45, 95%CI: 1.08–1.95, *p* = 0.0126), and moderate or severe kidney disease (sHR = 1.24, 95%CI: 1.08–1.43, *p* = 0.0030). Peptic ulcer disease, however, was associated with a reduced risk of sICH (sHR = 0.82, 95%CI: 0.74–0.90, *p* < 0.0001). Comorbidities that were not found to be independent risk factors were myocardial infarction, congestive heart failure, peripheral vascular disease, dementia, chronic lung disease, connective tissue disease, chronic liver disease, diabetes, hematologic malignancies, moderate or severe liver disease, and metastatic malignancies. There were not enough data to determine the significance of the association between comorbid AIDS and sICH. 

### 3.3. Statin Use and All-Cause Mortality

The hazard ratio for all-cause mortality was calculated using the Cox proportion model (Table 3). A lower mortality risk was found in the statin cohort (HR = 0.75, 95%CI: 0.73–0.76, *p* < 0.0001). A greater risk of mortality was found in older patients (HR = 1.07, 95%CI: 1.07–1.07, *p* < 0.0001) and male patients (HR = 1.28, 95%CI: 1.25–1.30, *p* < 0.0001). Patients treated with SSRIs had a higher risk of mortality (HR = 1.03, 95%CI: 1.01–1.05, *p* = 0.0057), and those treated with antihypertensives and NSAIDs had a lower risk (HR = 0.96, 95%CI: 0.94–0.99, *p* = 0.0142; HR = 0.84, 95%CI: 0.82–0.86, *p* < 0.0001, respectively).

Analyzing the mortality risk associated with the different comorbid diseases, we found a greater risk of mortality in those with myocardial infarction (HR = 1.11, 95%CI: 1.07–1.15), congestive heart failure (HR = 1.39, 95%CI: 1.35–1.43), peripheral vascular disease (HR = 1.21, 95%CI: 1.15–1.27), cerebrovascular disease (HR = 1.05, 95%CI: 1.02–1.07), dementia (HR = 1.24, 95%CI: 1.19–1.30), diabetes (HR = 1.36, 95%CI: 1.33–1.40), diabetes with end organ damage (HR = 1.35, 95%CI: 1.31–1.39), hemiplegia (HR = 1.32, 95%CI: 1.22–1.41), moderate or severe kidney disease (HR = 1.40, 95%CI: 1.36–1.45), hematologic malignancies (HR = 1.20, 95%CI: 1.15–1.25), moderate or severe liver disease (HR = 1.89, 95%CI: 1.54–2.31), and metastatic malignancies (HR = 1.65, 95%CI: 1.49–1.83) (all *p* < 0.0001). However, we found a decreased risk of mortality in those with chronic lung disease (HR = 0.98, 95%CI: 0.95–<1.00, *p* < 0.0263), peptic ulcer disease (HR = 0.91, 95%CI: 0.89–0.93, *p* < 0.0001), and chronic liver disease (HR = 0.90, 95%CI: 0.88–0.93, *p* < 0.0001). 

**Figure 3:**
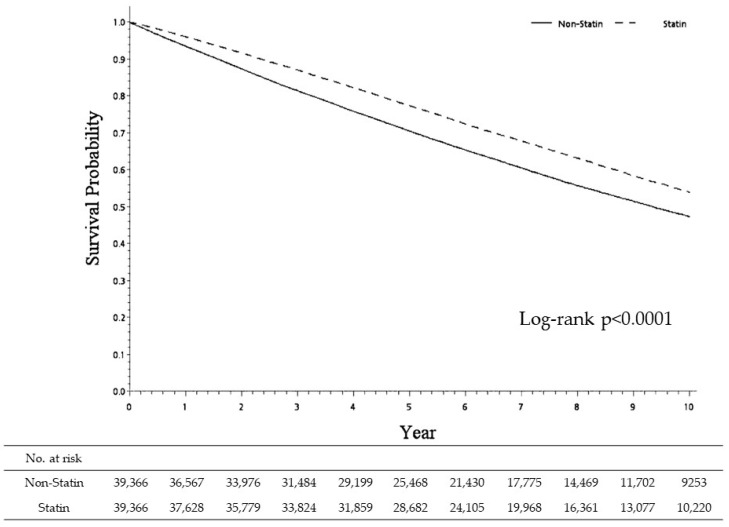
Survival probability between statin and non-statin cohorts for patients receiving statin treatment after ischemic stroke.

As seen in Figure 3, which shows the results of our survival probability analysis, patients prescribed statin were more likely to still be alive at the end of our ten-year study period (10,220 living statin users vs. 9253 living non-statin users; log-rank *p* < 0.0001), after adjusting for other medications and comorbidities. This finding is notable because they were at higher risk for sICH (sHR = 0.74; Table 2) and all-cause mortality (HR=0.75; Table 3).

### 3.4. Statin Dosage and Outcomes

Irrespective of dosage, statin users had a lower risk of sICH and all-cause mortality risk. Moreover, the higher the statin dose, the lower the incidence of sICH (low-dose: adjusted sHR = 0.82, 95%CI 0.73–0.92, *p* = 0.001, moderate-dose: adjusted sHR = 0.74, 95%CI 0.67–0.82, *p* < 0.0001, high-dose: adjusted sHR = 0.53, 95%CI 0.43–0.66, *p* < 0.0001) and the lower the all-cause mortality risk (low-dose: adjusted sHR = 0.75, 95%CI 0.73–0.78, *p* < 0.0001, moderate-dose: sHR = 0.74, 95%CI 0.73–0.76, *p* < 0.0001, high-dose: sHR = 0.74, 95%CI 0.71–0.78, *p* < 0.0001).

## 4. Discussion

In this study of the possible association between statin pharmacotherapy and risk of sICH and all-cause mortality in a nationwide cohort of AIS patients 50 years old and older, we found that statin use decreased the incidence of sICH and all-cause mortality, with the higher the dose, the greater the benefit. Recently, controversy regarding the safety of statin use after an AIS stems from the pleiotropic effects of statins on hemostasis and coagulation [11], dose-dependent antiplatelet [12], and antithrombotic activity [13]. This concern was highlighted in the SPARCL trial [7], where patients treated with atorvastatin after an AIS had a higher incidence of hemorrhagic stroke. The current study of a nationwide cohort in Taiwan did not corroborate their findings. In fact, it found that the higher the statin dose, the lower the risk of sICH (sHR 0.82, 0.74, and 0.53, respectively).

### 4.1. Statin Use and Subsequent ICH

This study found a reduced risk of sICH in AIS patients taking statins. However, one meta-analysis concluded that there was an increased risk of sICH in AIS patients taking statins in all the studies they analyzed except for one study conducted in Japan [4]. The rest of the studies they analyzed were conducted in Western countries. Our study and one population-based retrospective study evaluating the risk of sICH in pre-stroke and post-stroke statin users in Taiwan have found a decreased risk of sICH in AIS patients taking statins [14]. The reason for the difference in risk between our studies and those conducted previously needs further study. 

### 4.2. Statin Use and All-Cause Mortality

Meta-analyses by Judge, McKinney, and Cheng have all found a significant association between lipid-lowering pharmacotherapy and decreases in all-cause mortality [5,6,15]. In addition, a one-year mortality analysis by Lin et al. has reported the mortality rate to be highest in AIS patients not using statins (16.3%) [14]. They observed significantly lower mortality rates in their pre-stroke statin group (6.8%, HR = 0.56, 95%CI = 0.53–0.41, *p* < 0.0001) and their post-stroke statin group (5.4%, HR = 0.51, 95%CI = 0.48–0.53, *p* < 0.0001) compared to the non-statin group. In a retrospective cohort study of patients collected in one database, Schietz found that both patients taking statins pre-stroke and patients initiating statin use early after an AIS had lower short-term mortality rates than non-statin users [16]. Although we found a higher all-cause mortality rate in male patients, Rexrode et al. found that females had a high incidence of stroke-related mortality [17]. Whether this difference is related to statin use remains unclear. 

### 4.3. Other Medications and Comorbidities versus Outcomes

Other pharmacotherapeutic strategies and comorbidities can influence a patient’s sICH risk. For example, anticoagulants are known to interfere with the clotting cascade, increasing the risk of sICH [18]. As expected, our study also found that anticoagulants increased this risk (sHR 1.45, Table 2). However, we did not find that the use of antihypertensive agents, NSAIDs, or SSRIs increased the risk of sICH. We also found significant associations between many comorbidities and sICH. The same factors known to predispose patients to the initial cerebrovascular insult, which include hypertension, diabetes, and hyperlipidemia, also put them at greater risk of subsequent insult [19]. Poor glycemic control causes glycation and atherosclerosis, damaging blood vessels and thus increasing the risk of ICH [20,21], and damage in the form of moderate or severe kidney diseases can lead to ICH via uremic platelet dysfunction [22]. Uremia affects platelet function and coagulation factors, impairing platelet adhesion and aggregation, increasing bleeding time, and predisposing patients to ICH [23].

This study found some associations between specific comorbidities and our outcomes that we could not explain. For instance, we found a significant association between peptic ulcer disease and decreased risk of sICH and all-cause mortality after AIS (sHR 0.82 and 0.91, respectively) (Table 2 and Table 3). We could not find a reason for this inverse relationship. We also found a significant association between hemiplegia and increased risk of sICH and all-cause mortality. Although hemiplegia may not directly lead to ICH, it can increase the risk of falls and trauma, which can, in turn, lead to ICH. We did not find an association between chronic liver disease or moderate or severe liver disease and sICH. However, we did find a reduction in all-cause mortality in patients with chronic liver disease but an increase in all-cause mortality in those with moderate or severe liver disease. Typically, liver dysfunction affects the production and clearance of clotting factors, leading to a reduction in clotting and an elevation in fibrinolysis [24], making this reduction in all-cause mortality unclear.

### 4.4. Statin Dosage and Risks

This study found that the greater the statin dose, the lower the sICH risk in patients taking these drugs after initial AIS. The Cox regression results of another NHIRD study of statin dosage conducted in Taiwan found high-dose and medium-dose users to be at 0.49 and 0.45 the risk ICH, respectively, compared to low-dose users [25], a finding similar to ours. Taiwan’s population is 95% Han Chinese. It would be interesting to have a large prospective study of another Han Chinese population. Thus, we look forward to the results of the CHRISTMAS study protocol, a multicenter, prospective, randomized control trial for patients receiving statins after an AIS in China [26]. Their findings may provide more insight into the association between statin use after an AIS and sICH.

It should be noted that several other studies have not produced consistent findings on the effect of statin dosage. One retrospective cohort did not find a significant difference between high-dose or low-dose statin usage and sICH [27], while another study found no relationship between statin dosage and all-cause mortality [28]. 

There have been studies suggesting that statin pharmacotherapy after ischemic stroke might increase the risk of sICH because low cholesterol levels may lead to fragile vascular endothelium and arterial vulnerability, hemorrhage, or lengthier repair after intracerebral microbleeds [12], especially in uncontrolled hypertension [29]. It has also been suggested that fragile vascular endothelial tissue may be more susceptible to microaneurysms [12]. Still, another study has proposed that statins may impede platelet aggregation and enhance fibrinolysis, resulting in antithrombotic activity [30].

Real-world studies of this association have not been in agreement. On the one hand, some have found an association between statin use and increased risk of stroke. One meta-analysis of trial studies by Judge et al. found a higher risk of ICH in those taking low-density lipoprotein-lowering medications for secondary prevention; though based on their number needed to harm and number needed to treat calculations, the benefits of lipid-lowering pharmacotherapy in preventing an AIS far exceeds the risk of ICH [5]. Likewise, a meta-analysis by Cheng et al. also reported a significantly elevated risk of ICH in patients receiving statin pharmacotherapy [6]. The Treat Stroke to Target trial found an insignificant increased risk of ICH in their <70 mg/dL treatment group compared to their 100 ± 10 mg/dL treatment group [31]. On the other hand, several studies have not found patients receiving statin pharmacotherapy to have a significant difference in the risk of sICH [5,15,27,28,32].

### 4.5. Limitations

This study has some limitations. One limitation is that it is a retrospective study, so selection bias cannot be avoided. Another limitation is that the NHIRD does not contain records on functional outcomes, including disability and dependence. Furthermore, unlike hospital medical records, the NHIRD does not collect or maintain patients’ lipid profiles. Although we found high-dose statin treatment to be most effective at lowering the sICH rate or all-cause mortality risk, the prescription of high doses requires dosage titration. Therefore, the lack of lipid profiles and their potential correlation with sICH rate or all-cause mortality could not be examined.

## 5. Conclusions

This nationwide real-world study found that statin use after an AIS can significantly lower the risk of sICH and all-cause mortality. Furthermore, the higher the statin dosage, the lower the sICH risk and all-cause mortality. Future prospective studies, including patients’ functional outcomes, lipid profiles, and prescribed statin doses, can be performed to obtain more conclusive results.

## Figures and Tables

**Table 1:** Baseline characteristics of the study population.

	Non-Statin Cohort N = 39,366	Statin Cohort N = 39,366	*p*-Value
Age	67.67 ± 9.09	68.16 ± 9.24	<0.0001
Gender			>0.9999
Female	17,898 (45.47)	17,898 (45.47)	
Male	21,468 (54.53)	21,468 (54.53)	
Statin dose			
Low		12,318 (31.29)	
Middle		21,729 (55.20)	
High		5319 (13.51)	
Statin (average days)			
Low		294.84 ± 77.72	
Middle		242.70 ± 112.38	
High		209.32 ± 129.71	
Medication			
Thrombolytic agents	<3 (<1) ^a^	<3 (<1) ^a^	
Anticoagulant agents	2540 (6.45)	2476 (6.29)	0.3504
Antihypertensive agents	33,810 (85.89)	33,608 (85.37)	0.0401
Non-steroidal anti-inflammatory drugs	26,634 (67.66)	26,842 (68.19)	0.1123
Selective serotonin reuptake inhibitors	16,668 (42.34)	17,038 (43.28)	0.0077
Comorbidities			
Myocardial infarct	2989 (7.59)	3559 (9.04)	<0.0001
Congestive heart failure	4509 (11.45)	4774 (12.13)	0.0034
Peripheral vascular disease	1513 (3.84)	1635 (4.15)	0.0265
Cerebrovascular disease	17,688 (44.93)	18,451 (46.87)	<0.0001
Dementia	1351 (3.43)	1606 (4.08)	<0.0001
Chronic lung disease	12,965 (32.93)	13,507 (34.31)	<0.0001
Connective tissue disease	998 (2.54)	925 (2.35)	0.0919
Ulcer	14,147 (35.94)	14,402 (36.58)	0.0587
Chronic liver disease	6403 (16.27)	5883 (14.94)	<0.0001
Diabetes	17,275 (43.88)	17,524 (44.52)	0.074
Diabetes with end organ damage	6943 (17.64)	7147 (18.16)	0.0579
Hemiplegia	602 (1.53)	605 (1.54)	0.9307
Moderate or severe kidney disease	4055 (10.30)	4276 (10.86)	0.0105
Tumor, leukemia, lymphoma	2626 (6.67)	2608 (6.63)	0.7968
Moderate or severe liver disease	72 (0.18)	73 (0.19)	0.9338
Malignant tumor, metastasis	319 (0.81)	290 (0.74)	0.2381
Acquired immunodeficiency syndrome	3 (0.01)	3 (0.01)	>0.9999
Intracerebral hemorrhage	1238 (3.14)	981 (2.49)	<0.0001
Death	20,371 (51.75)	17,620 (44.76)	<0.0001

^a^: Precise data not available to protect patient confidentiality.

**Table 2:** Predictive factors of subsequent intracerebral hemorrhage.

	Crude	*p*-Value	Adjusted	*p*-Value
	sHR ^a^		sHR ^a^	
Statin vs. non-statin	0.74 (0.68–0.81)	<0.0001	0.74 (0.68–0.80)	<0.0001
Age	1.00 (0.99–1.00)	0.5141	1.00 (0.99–1.00)	0.8965
Male vs. female	1.07 (0.99–1.17)	0.0963	1.07 (0.98–1.16)	0.1395
Medication				
Thrombolytic agents ^b^	omitted		omitted	
Anticoagulant agents	1.49 (1.28–1.72)	<0.0001	1.45 (1.24–1.70)	<0.0001
Antihypertensive agents	1.04 (0.92–1.18)	0.5109	1.03 (0.91–1.16)	0.6602
Non-steroidal anti-inflammatory drugs	0.87 (0.80–0.95)	0.0024	0.92 (0.84–1.01)	0.0992
Selective serotonin reuptake inhibitors	0.96 (0.88–1.04)	0.3179	0.98 (0.90–1.06)	0.5854
Comorbidities				
Myocardial infarct Congestive heart failure Peripheral vascular disease Cerebrovascular disease Dementia Chronic lung disease Connective tissue disease Ulcer Chronic liver disease Diabetes Diabetes with end organ damage Hemiplegia Moderate or severe kidney disease Tumor, leukemia, lymphoma Moderate or severe liver disease Malignant tumor, metastasis Acquired immunodeficiency syndrome ^c^	0.88 (0.74–1.04) 1.02 (0.88–1.17) 1.08 (0.87–1.36) 1.17 (1.07–1.27) 1.05 (0.82–1.34) 0.96 (0.88–1.05) 0.78 (0.57–1.07) 0.84 (0.77–0.92) 1.01 (0.90–1.14) 1.06 (0.97–1.15) 1.20 (1.08–1.34) 1.55 (1.15–2.07) 1.25 (1.09–1.43) 1.08 (0.91–1.29) 0.62 (0.16–2.48) 0.90 (0.50–1.62) NA ^c^	0.1209 0.8098 0.4771 0.0003 0.6939 0.3743 0.1210 0.0002 0.8096 0.1880 0.0009 0.0035 0.0014 0.3748 0.5016 0.7148	0.87 (0.73–1.04) 0.99 (0.85–1.15) 1.06 (0.84–1.32) 1.18 (1.08–1.28) 1.03 (0.81–1.33) 0.97 (0.89–1.07) 0.80 (0.59–1.10) 0.82 (0.74–0.90 1.03 (0.91–1.16) 1.00 (0.91–1.10) 1.19 (1.05–1.35) 1.45 (1.08–1.95) 1.24 (1.08–1.43) 1.11 (0.92–1.33) 0.65 (0.16–2.60) 0.83 (0.45–1.54) NA ^c^	0.1206 0.8657 0.6410 0.0002 0.7969 0.5991 0.1685 <.0001 0.6429 0.9809 0.0067 0.0126 0.0030 0.2748 0.5412 0.5590

^a^: subdistribution hazard ratio. ^b^: thrombolytics removed due to omitted results. ^c^: statistical analysis not available due to small sample size.

**Table 3:** Predictive factors of all-cause mortality.

	Crude	*p*-Value	Adjusted	*p*-Value
	HRs ^a^		HRs ^a^	
Statin vs. non-statin	0.81 (0.80–0.83)	<0.0001	0.75 (0.73–0.76)	<0.0001
Age	1.07 (1.07–1.07)	<0.0001	1.07 (1.07–1.07)	<0.0001
Male vs. female	1.01 (0.99–1.03)	0.4933	1.28 (1.25–1.30)	<0.0001
Medication				
Antithrombotic agents	2.38 (0.90–6.34)	0.0813	1.93 (0.72–5.14)	0.1885
Anticoagulant agents	1.14 (1.10–1.19)	<0.0001	1.03 (0.98–1.07)	0.2420
Antihypertensive agents	1.05 (1.02–1.08)	0.0009	0.96 (0.94–0.99)	0.0142
Non-steroidal anti-inflammatory drugs	0.78 (0.76–0.79)	<0.0001	0.84 (0.82–0.86)	<0.0001
Selective serotonin reuptake inhibitors	1.06 (1.04–1.09)	<0.0001	1.03 (1.01–1.05)	0.0057
Comorbidities Myocardial infarct Congestive heart failure Peripheral vascular disease Cerebrovascular disease Dementia Chronic lung disease Connective tissue disease Ulcer Chronic liver disease Diabetes Diabetes with end organ damage Hemiplegia Moderate or severe kidney disease Tumor, leukemia, lymphoma Moderate or severe liver disease Malignant tumor, metastasis Acquired immunodeficiency syndrome	1.49 (1.44–1.54) 1.96 (1.90–2.01) 1.61 (1.54–1.69) 1.29 (1.26–1.32) 2.21 (2.11–2.31) 1.36 (1.34–1.39) 1.16 (1.09–1.24) 1.21 (1.19–1.24) 1.01 (0.99–1.04) 1.54 (1.51–1.58) 1.74 (1.70–1.78) 1.52 (1.41–1.63) 1.91 (1.86–1.97) 1.66 (1.60–1.72) 1.90 (1.55–2.33) 2.19 (1.98–2.42) 0.93 (0.23–3.69)	<0.0001 <0.0001 <0.0001 <0.0001 <0.0001 <0.0001 <0.0001 <0.0001 0.3458 <0.0001 <0.0001 <0.0001 <0.0001 <.00001 <0.0001 <0.0001 0.9124	1.11 (1.07–1.15) 1.39 (1.35–1.43) 1.21 (1.15–1.27) 1.05 (1.02–1.07) 1.24 (1.19–1.30) 0.98 (0.95-<1.00) 0.98 (0.92–1.04) 0.91 (0.89–0.93) 0.90 (0.88–0.93) 1.36 (1.33–1.40) 1.35 (1.31–1.39) 1.32 (1.22–1.41) 1.40 (1.36–1.45) 1.20 (1.15–1.25) 1.89 (1.54–2.31) 1.65 (1.49–1.83) 0.79 (0.20–3.17)	<0.0001 <0.0001 <0.0001 <0.0001 <0.0001 0.0263 0.5019 <0.0001 <0.0001 <0.0001 <0.0001 <0.0001 <0.0001 <0.0001 <0.0001 <0.0001 0.7416

^a^: hazard ratios.

## Data Availability

Details regarding the database generation, monitoring, and maintenance are provided on the NHIRD website (http://nhird.nhri.org.tw/) (accessed 1 August 2019).

## Supplementary Materials

The following supporting information can be downloaded at: https://www.mdpi.com/article/10.3390/medicina60060939/s1, Table S1: Charlson Comorbidity Index and its respective ICD-9-CM codes.
