# Supplementary material for: Nationwide Big Data Analysis of Statin Use and Intracerebral Hemorrhage Risk in Acute Ischemic Stroke Patients in Taiwan
Source: Medicina (Kaunas). 2024 Jun 4;60(6):939. doi: 10.3390/medicina60060939 (PMC11205390; doi:10.3390/medicina60060939)
Supplement: Supplementary file 1 [file medicina-60-00939-s001.zip › medicina-3031373-supplementary.pdf]

Table S1. Charlson Comorbidity Index and its Respective ICD-9-CM codes.

Comorbidity	ICD-9-CM
Myocardial infarction	410.X and 412.X
Congestive heart failure	428.X
Peripheral vascular disease	443.9, 441.X, 785.4, V434, 384.8
Cerebrovascular disease	430.X-438.X
Dementia	290.X
Chronic lung disease	490.X-496.X, 500.X-505.X, and 506.4
Connective tissue disease	710.0, 710.1, 710.4, 714.0-714.2, 714.81, 725.X
Ulcerative disease	531.X-534.X
Chronic liver disease	571.2, 571.4, 571.5, and 571.6
Diabetes	250.0-250.3, 250.7
Diabetes with end-organ damage	250.4-250.6
Hemiplegia	344.1, 342.X
Moderate or severe kidney disease	582.X, 583.0-583.7, 585.X-586.X, 588.X
Leukemia or lymphoma-related tumors	140.X-195.X, 200.X-208.X
Moderate or severe liver disease	572.2-572.8, and 456.00-456.21
Malignant tumor/metastasis	196.0-199.1
Acquired immunodeficiency syndrome	042.0-044.9
